# Perioperative Outcomes of No-Drain Strategy in Primary Repair of Perforated Peptic Ulcer: A Systematic Review and Meta-Analysis

**DOI:** 10.3390/medicina62051003

**Published:** 2026-05-21

**Authors:** Lorenzo Dell’Atti, Maurizio Zizzo, Andrea Morini, Federica Mereu, Marco Scarpa, Quoc Riccardo Bao, Silvia Negro, Emanuele Damiano Luca Urso, Dario Parini, Massimiliano Fabozzi

**Affiliations:** 1 Surgical Oncology Unit, Azienda Unità Sanitaria Locale—IRCCS di Reggio Emilia, 42123 Reggio Emilia, Italy; maurizio.zizzo@ausl.re.it (M.Z.); andrea.morini@ausl.re.it (A.M.); federica.mereu@ausl.re.it (F.M.); massimiliano.fabozzi@ausl.re.it (M.F.); 2 General Surgery, 3rd Division, Padua University Hospital, 35128 Padua, Italy; marco.scarpa@unipd.it (M.S.); silvia.negro@unipd.it (S.N.); edl.urso@unipd.it (E.D.L.U.); 3 General Surgery Unit, Ospedale Santa Maria della Misericordia, AULSS 5 Polesana, 45100 Rovigo, Italy; dario.parini@aulss5.veneto.it

**Keywords:** perforated peptic ulcer, drain omission, Graham repair, leak

## Abstract

*Background and Objectives:* Perforated peptic ulcer (PPU) is an emergent condition managed by surgical intervention. No conclusive evidence has been produced regarding the need for drain placement after primary repair. Our meta-analysis aimed to provide insight into the short-term outcomes by comparing the two strategies of drain omission or intra-operative placement of at least one drain. *Materials and Methods*: We performed a systematic review following the PRISMA guidelines. PubMed/MEDLINE, Web of Science, Cochrane Library, and Embase databases were utilized to identify articles of interest. Meta-analysis was performed using RevMan Version 5.4. Eligible studies were comparative studies (RCTs and observational studies) enrolling adult patients (≥18 years) undergoing emergency primary repair for PPU, with or without prophylactic intra-abdominal drain placement; case reports and series of fewer than 10 patients were excluded. The literature search covered January 2010 to 22 February 2026. Risk of bias was assessed using the Cochrane RoB 2.0 tool for RCTs, and the ROBINS-I V2 tool for observational studies; certainty of evidence was graded using the GRADE framework. Pooled effect estimates were calculated using a random-effects model and expressed as odds ratios (OR) or mean differences (MD) with 95% confidence intervals (CI); statistical heterogeneity was quantified using the I^2^ statistic. *Results*: Five studies were considered for comparison, for a total of 1354 patients (744 and 610 in the drain and no-drain groups, respectively). Three were randomized controlled trials, and two were retrospective cohort studies, conducted across four countries (India, the USA, Egypt, and Japan). Meta-analysis of the pooled results showed that drain omission was associated with a shorter length of stay (LOS) (MD −2.13, 95% CI [−3.91–−0.35], *p* < 0.0001) and a lower rate of superficial surgical site infections (SSIs) (16.7% vs. 52.7%, OR 0.24, 95%CI [0.11–0.55], *p* = 0.0007). No difference was observed regarding the rate of leaks, reoperation, or deep SSIs. Low-certainty evidence suggested higher postoperative mortality in the no-drain group (OR: 1.96; 95% CI: 1.10 to 3.48; *p* = 0.02; I^2^ = 0%), largely driven by retrospective studies with a high risk of bias. This mortality finding is of very low certainty and is most likely attributable to confounding in the observational studies rather than a true causal effect of drain omission. Several outcomes were based on data from only two to three studies, and the overall certainty of evidence was low to very low. *Conclusions*: Drain omission after primary repair for PPU may be associated with better outcomes in terms of LOS and superficial SSIs, primarily in lower-acuity patients, as reflected by the inclusion criteria of the contributing RCTs. Pooled analysis showed a higher postoperative mortality in the no-drain group; however, given the significant biases among included studies, our results should be interpreted as non-causal and thus require careful interpretation. Further research encompassing the full clinical spectrum of PPU is needed to confirm our results. Evidence certainty was low to very low across all outcomes, primarily due to a risk of bias, high heterogeneity (I^2^ up to 95% for LOS), indirectness, and imprecision.

## 1. Introduction

Peptic ulcer disease (PUD) remains a significant global health concern, affecting millions of individuals annually and contributing substantially to healthcare costs and morbidity [1]. The pathophysiology of PUD is primarily driven by an imbalance between aggressive factors, such as gastric acid and pepsin, and mucosal defensive mechanisms.

The advent of proton pump inhibitors (PPIs) and effective eradication therapies for *Helicobacter pylori* has led to a shift from primary surgical to predominantly medical management. These therapeutic strategies boast high success rates, effectively healing ulcers and preventing recurrence in most patients [2]. However, despite these advances, the rate of complicated PUD, which includes bleeding, perforation, and gastric outlet obstruction, has not declined dramatically [3,4].

Perforation is one of the most feared complications of PUD, representing a surgical emergency with a reported incidence of 2–14% in adult patients with PUD and carrying a mortality rate that can range from 10% to 25% [5,6,7]. These figures pertain to the adult population; in the pediatric and adolescent population, PUD and its complications are considerably less common, with a reported incidence of approximately 1–2% and a substantially lower associated mortality of around 1–4% [8]. A perforated peptic ulcer (PPU) causes a leak of gastroduodenal contents into the peritoneal cavity, resulting in chemical peritonitis, which then rapidly progresses to bacterial peritonitis and sepsis if left untreated.

The mainstay of treatment for PPU is surgical intervention, with the primary goals of source control and resuscitation [5]. The standard surgical procedure is primary repair of the perforation, often reinforced with an omental patch (a Graham patch), which can be performed via laparotomy or laparoscopy. While both approaches are effective, the choice of technique often depends on the patient’s stability, the surgeon’s expertise, and the size and location of the ulcer [9].

However, the postoperative course for patients with PPU is often accompanied by complications, including surgical site infections (SSIs) and prolonged ileus, which contribute to significant morbidity and extended hospital stays [10].

For decades, the routine placement of prophylactic intra-abdominal drains following PPU repair was considered non-negotiable. The theoretical advantages of drainage included the evacuation of residual contaminated fluids, the creation of a controlled fistula in case of leak of the repair, and the early detection of postoperative hemorrhage or anastomotic leakage. However, this traditional practice has been increasingly challenged. Drains are foreign bodies that can themselves serve as a nidus for infection, provoke an inflammatory response, cause significant patient discomfort, and potentially prolong hospitalization [11]. Furthermore, accumulating evidence from other abdominal surgeries (including pancreatic and colorectal surgery) has questioned the benefit of routine drainage, suggesting that it may be associated with increased complication rates without conferring the intended advantages [12,13].

While individual studies have investigated the role of drains in the context of PPU repair, their findings have been limited by small sample sizes and heterogeneous patient populations, leading to a lack of consensus and persistent variability in surgical practice. To date, no comprehensive synthesis of the available evidence has been performed to definitively evaluate the impact of a no-drain strategy on perioperative outcomes in patients undergoing primary repair for PPU.

Therefore, we conducted a systematic review and meta-analysis of the available literature. The objective of this study was to compare the perioperative outcomes of a no-drain strategy versus routine drain placement in patients undergoing primary repair for PPU, with a focus on morbidity, mortality, and recovery metrics.

## 2. Materials and Methods

### 2.1. Study Registration

This systematic review and meta-analysis were conducted in accordance with the Preferred Reporting Items for Systematic Reviews and Meta-Analyses (PRISMA) guidelines [14]. The review protocol was prospectively registered on the International Prospective Register of Systematic Reviews (PROSPERO) with the registration number CRD420251053099, effective on 14 May 2025. The full review protocol is publicly accessible via the PROSPERO registry (https://www.crd.york.ac.uk/prospero/display_record.php?RecordID=1053099 (accessed on 15 April 2026)). No amendments to the pre-registered protocol were made following registration.

### 2.2. Search Strategy

A comprehensive literature search was performed to identify all relevant studies comparing drain omission versus drain placement strategies in patients undergoing primary repair for perforated peptic ulcer. We systematically searched the electronic databases MEDLINE/PubMed, Web of Science, Cochrane Library, and Scopus for studies published between 1 January 2010 and 22 February 2026 without additional restrictions. The 2010 lower boundary was selected to capture evidence reflecting contemporary surgical practice, as drain omission strategies in PPU repair have largely emerged as a clinical question in the context of modern perioperative care and the wider adoption of laparoscopic repair, which became more prevalent from approximately 2005 onwards. Studies conducted in earlier eras under substantially different anesthetic and postoperative care standards may not be directly relevant to current practice. The latest electronic search was performed on 22 February 2026. The article screening and article selection process was performed using the Rayyan software for systematic reviews and meta-analyses [15]. Two reviewers (LDA and AM) independently reviewed titles and abstracts of all the studies retrieved by the search strategy. A third reviewer (MZ) resolved any conflicts. To ensure literature saturation, we manually screened the reference lists of all included studies for additional potentially eligible studies (snowballing).

We used the following queries:

• MEDLINE/Pubmed: (“peptic ulcer perforation”[MeSH Terms] OR “perforated peptic ulcer”[Title/Abstract] OR “PPU”[Title/Abstract] OR “duodenal ulcer perforation”[Title/Abstract]) AND (“drainage”[MeSH Terms] OR “drain*”[Title/Abstract] OR “no drain”[Title/Abstract] OR “abdominal drain”[Title/Abstract]) AND (“surgical procedures, operative”[MeSH Terms] OR “surgical repair”[Title/Abstract] OR “primary repair”[Title/Abstract] OR “oment* patch”[Title/Abstract] OR “graham patch”[Title/Abstract] OR “closure”[Title/Abstract]);
• Cochrane Library: TS=(((“peptic ulcer” OR “duodenal ulcer”) NEAR/3 perforat*) OR “PPU”) AND TS=(drain* OR “no drain” OR drainage) AND TS=(“surgical repair” OR “primary repair” OR “oment* patch” OR “graham patch” OR closure);
• Web of science: (“peptic ulcer perforation” OR “perforated peptic ulcer” OR “PPU”):ti,ab,kw AND (drain* OR drainage):ti,ab,kw;
• Scopus: TITLE-ABS-KEY((“peptic ulcer” W/3 perforat*) OR “perforated peptic ulcer” OR “PPU” OR “duodenal ulcer perforation”) AND TITLE-ABS-KEY(drain* OR “no drain” OR drainage) AND TITLE-ABS-KEY(“surgical repair” OR “primary repair” OR “oment* patch” OR “graham patch” OR closure).

### 2.3. Inclusion Criteria

All comparative studies reporting clinical outcomes of patients who underwent primary surgical repair with or without abdominal drain for perforated peptic ulcer were considered eligible for inclusion in this systematic review and meta-analysis. Randomized controlled trials (RCTs), controlled clinical trials, cohort studies, case series, and case–control studies were considered eligible. Articles in languages other than English were excluded. Editorials, review articles, invited commentaries, case reports, and studies with <10 cases were excluded. Gray literature (i.e., conference abstracts, trial registries, or institutional repositories) was not included for screening. In the case of studies reporting overlapping cohorts, the most recent study was considered for inclusion.

We present our inclusions in compliance with PICOs criteria:

• P (population): Adult patients (≥18 years) undergoing emergency primary surgical repair for perforated peptic ulcer (gastric or duodenal).
• I (intervention): Primary repair of the perforation (with or without omental patch) performed via laparotomy or laparoscopy without the placement of a prophylactic intra-abdominal drain.
• C (comparators): Primary repair of the perforation with the placement of at least one prophylactic intra-abdominal drain.
• O (outcomes): Studies reporting at least one of the pre-specified outcomes listed below.
• S (study designs): RCTs and observational studies (cohort studies, case–control studies) were considered for inclusion. Case reports, case series with fewer than 10 patients, conference abstracts, editorials, expert opinions, and animal studies were excluded.

### 2.4. Outcomes

The outcomes of interest were selected based on their clinical relevance to postoperative recovery and their consistent reporting across the included studies.

Primary Outcomes:

• Length of hospital stay (LOS): Defined as the total duration of hospitalization, measured in days from the date of admission to the date of hospital discharge, serving as a marker of the overall uneventfulness of postoperative recovery.
• Leak: Defined as failure of the surgical closure resulting in extravasation of intraluminal contents, confirmed by imaging (computed tomography with oral contrast, upper gastrointestinal series) or direct visualization at reoperation, according to each study protocol.

Secondary Outcomes:

To provide a comprehensive assessment of the safety and efficacy of the no-drain strategy, the following secondary outcomes were evaluated:

• Postoperative morbidity: The proportion of patients experiencing any complication during index hospitalization. Complications were categorized according to the Clavien–Dindo classification, where reported, further focusing on major complications (Clavien–Dindo ≥ III).
• Mortality: 30-day mortality from any cause.
• SSIs: Superficial or deep incisional SSI occurring within 30 days post-surgery, defined according to the Centers for Disease Control and Prevention (CDC) criteria. Further analysis was planned to distinguish between superficial and deep SSIs, where feasible.
• Prolonged postoperative ileus: Defined as the failure to tolerate oral intake, the need for nasogastric tube reinsertion beyond postoperative day 5, or as defined by the individual study authors.
• Reoperation rate: The proportion of patients requiring unplanned reoperation for any cause.
• Drain-related complications: For the drain group of the included studies, complications directly attributable to the drain, including drain site infection, blockage, dislodgement, or persistent pain or discomfort requiring early drain removal or analgesics beyond routine postoperative care.

### 2.5. Data Extraction

Two review authors [LDA and AM] independently screened the titles and abstracts of all retrieved records against the inclusion criteria. Full texts of potentially relevant studies were obtained and independently assessed for eligibility by the same two authors. Any disagreements were resolved through discussion or by consultation with a third author [MZ].

For each eligible study, two authors independently extracted the following data using a predesigned extraction form and subsequently integrated:

• Study characteristics: First author, year of publication, country, study design, sample size, study period, and single- or multi-center setting.
• Patient characteristics: Age, sex, Boey score or AAST (American Association for the Surgery of Trauma) grade, American Society of Anesthesiologists (ASA) status classification, perforation site (gastric vs. duodenal), perforation size, time from perforation to surgery, and presence of shock at admission.
• Intervention details: Surgical approach (laparoscopic vs. open), type of repair (simple closure vs. omental patch), type of drain used, and drain management protocol.
• Outcome data: For each outcome of interest, the number of events in each group and the total number of patients analyzed, or the mean and standard deviation for continuous outcomes.

### 2.6. Assessment of Risk of Bias

Two review authors [LDA and AM] independently assessed the methodological quality and risk of bias of the included studies.

• For RCTs: The Cochrane Risk of Bias tool (RoB 2.0) was used [16]. Studies were judged as having “low risk,” “some concerns,” or “high risk” of bias across the following domains: randomization process, deviations from intended interventions, missing outcome data, measurement of the outcome, and selection of the reported result.
• For Observational Studies: The risk of bias in non-randomized studies of interventions was assessed using the Risk of Bias in Non-randomized Studies of Interventions (ROBINS-I) tool (version 2) [17]. Studies were judged as having “low risk of bias,” “moderate risk of bias,” “serious risk of bias,” or “critical risk of bias” across the following domains: bias due to confounding, bias in selection of participants into the study, bias in classification of interventions, bias due to deviations from intended interventions, bias due to missing data, bias in measurement of outcomes, and bias in a selection of the reported result.

Any disagreements in risk of bias assessment were resolved through discussion or arbitration by a third reviewer [MZ]. The results of the risk of bias assessment will be presented in summary tables and figures and will inform sensitivity analyses.

The certainty of evidence for each outcome was assessed using the Grading of Recommendations, Assessment, Development, and Evaluations (GRADE) tool [18]. Evidence from randomized controlled trials was initially considered high certainty, while evidence from observational and retrospective studies was initially considered low certainty. The certainty was rated down based on five factors: risk of bias, inconsistency, indirectness, imprecision, and publication bias. The final certainty for each outcome was categorized as high, moderate, low, or very low. A Summary of Findings (SoF) table was constructed using GRADEpro GDT software v. 3.0 to present the main results and certainty ratings in a transparent and accessible format.

### 2.7. Data Analysis

Statistical analysis of the collected data was performed using Review Manager 5.4 (Cochrane Collaboration, 2020). All data expressed in median and interquartile range (IQR) were converted into mean and standard deviation (SD) according to Wan formulas [19]. Mean and SD for multiple groups were combined into one according to the Cochrane formulae. A meta-analysis was performed if at least two studies reported data for a given outcome [20]. Given the anticipated clinical and methodological heterogeneity across studies, a random-effects model (DerSimonian and Laird method) was used for all primary analyses, as it provides a more conservative estimate by accounting for both within-study and between-study variance.

Statistical heterogeneity was assessed using the Cochran’s Q test (Chi^2^ test) and quantified using the I^2^ statistic. I^2^ values were interpreted as follows: < 25% low heterogeneity, 25–50% moderate heterogeneity, and >50% high heterogeneity. These thresholds align with established conventions while allowing for clinically meaningful interpretation.

When heterogeneity was low to moderate (I^2^ < 50%), a fixed-effects model was applied to the sensitivity analyses to assess the robustness of findings. When heterogeneity was high (I^2^ > 50%), the leave-one-out method (influence analysis) was used to assess the impact of individual studies on the overall pooled estimate, where heterogeneity was high. This involves sequentially removing one study at a time and recalculating the pooled effect to identify outliers or studies exerting undue influence on the results.

If sufficient studies were available, the following subgroup analyses were planned to explore potential sources of heterogeneity, performing separate analyses based on the following: study design (RCTs and non-RCTs), surgical approach (laparoscopic and open repair), perforation site (gastric and duodenal ulcers), and patient risk profile (high-risk patients, defined by a Boey score ≥2 or ASA ≥3, and low-risk patients).

Publication bias was assessed for outcomes reported by at least 10 studies using funnel plots. Asymmetry of the funnel plot was visually inspected and statistically tested using Egger’s linear regression test (*p* < 0.10 considered indicative of significant asymmetry).

For all pooled effect estimates, statistical significance was set at *p* < 0.05.

## 3. Results

### 3.1. Study Selection

**Figure 1:**
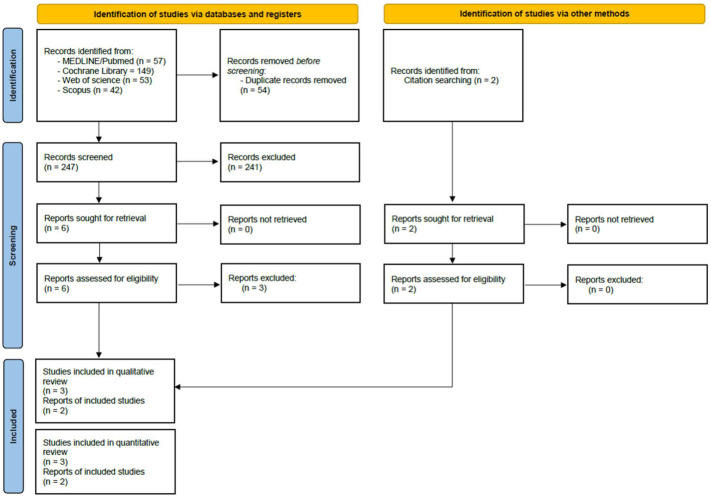
PRISMA flowchart of the literature search.

The literature search was conducted independently on each mentioned database, as shown in Figure 1. After a full-text assessment, duplicated studies were then excluded.

Our literature search provided a total of 301 studies. After screening, three studies were included for analysis: two retrospective cohort studies and one RCT [21,22,23]. After manually screening the reference lists of all included studies and available literature, we included two additional RCTs [24,25], for a total of five studies (three RCTs and two retrospective studies). Of the two selected retrospective studies, Okumura et al. provided analysis for the general cohort and a propensity-score matched (PSM) cohort; in order to lower potential selection bias, we decided to include only the PSM cohort in our analysis. Three studies were excluded after full-text review. The main reasons for exclusion were an absence of a comparator group and an absence of outcomes of interest.

### 3.2. Clinical Characteristics

A total of five studies comprising 1354 patients were included in this systematic review and meta-analysis. Among these, 744 patients (54.9%) underwent primary repair with prophylactic intra-abdominal drain placement, while 610 patients (45.1%) were managed with a no-drain strategy. The characteristics of the included studies are summarized in Table 1, Table 2 and Table 3.

Of the five included studies, three were RCTs and two were retrospective cohort studies. Four studies were conducted at single centers, while one large retrospective study was a multi-center analysis including 324 institutions across Japan. Geographically, two studies originated from India, one from the USA, one from Egypt, and one from Japan.

The sample sizes varied considerably, ranging from 60 to 934 patients. The largest study by Okumura et al. contributed 69% of the total patient population (934 patients). All studies included adult patients undergoing emergency surgery for a perforated peptic ulcer. Frequent exclusion criteria across studies were as follows: perforation size >2 cm, presence of frank pyoperitoneum or purulent peritonitis, delayed presentation beyond 48–72 h, hemodynamic instability or shock, ASA score III-IV, suspected malignancy or traumatic perforation.

All studies employed a primary repair with omental patch (Graham patch) as the standard surgical technique, with some studies also including a modified Graham patch or simple suturing. The surgical approach varied: three studies exclusively used open repair, while two studies included both open and laparoscopic approaches. Among studies reporting drain usage, Ansari et al. [24] reported three different groups according to drain number and site (sub-hepatic and/or pelvic), Nageeb et al. [22] reported the use of two drains (sub-hepatic and pelvic), Kumar et al. [25] used a 28 Fr drain; other studies did not specify drain characteristics.

The overall study period covered approximately 25 years (from 2008 to 2024). Patients were predominantly male (848, 71.9%) with a mean age between 39.2 and 45.08 years.

Only Ansari et al. reported the perforation site in detail. Among their 114 patients, the majority had duodenal perforations (90.5% and 94.7% in the drain and no-drain group, respectively), with gastric perforations comprising the remainder. The other four studies did not provide information about the perforation site.

Two studies reported perforation size. Ansari et al. [24] categorized size as <1 cm or 1–2 cm (patients with perforations >2 cm were excluded). Collectively in their drain groups, 82.1% had perforations <1 cm and 17.9% had perforations 1–2 cm; in the no-drain group, 16 (84.2%) had perforations <1 cm and 5 (15.8%) had perforations 1–2 cm. Nageeb et al. [22] reported the mean perforation size, which was nearly identical between groups: 8.75 ± 3.85 mm versus 8.71 ± 3.90 mm in the drain and no-drain groups, respectively. Both studies excluded patients with perforations >2 cm, ensuring a relatively homogeneous population with respect to this important prognostic factor.

Delayed presentation is a well-established risk factor for poor outcomes in PPU [26,27]. Ansari et al. reported time to surgery in hours: in the no-drain vs. drain group, respectively, 15.8 vs. 15.8% presented within 0–24 h, 68.4 vs. 69.5% within 24–48 h, and 15.8 vs. 14.7% within 48–72 h. The other studies did not report this variable in a format suitable for comparison.

Ansari et al. quantified contamination by volume: in the no-drain and drain groups, respectively, 73.7 vs. 76.8% had <500 mL, 10.5 vs. 8.4% had 500–1000 mL, and 15.8 vs. 14.7% had >1000 mL. Nageeb et al. [22] also reported contamination volume: in the no-drain and drain groups, respectively, 57.1 vs. 44.4% had <500 mL, 34.3 vs. 44.4% had 500–1000 mL, and 8.6 vs. 11.1% had >1000 mL. The remaining studies did not report this variable.

Only Okumura et al. [23] reported the presence of shock at presentation, reporting this condition in 9.2% in the drain group and 9.6% in the no-drain group. The other studies either excluded hemodynamically unstable patients or did not report this variable.

Reporting of validated severity scores was inconsistent across studies. Nageeb et al. [22] explicitly excluded patients with ASA III–IV, limiting their population to lower-risk surgical candidates; the remaining studies did not report ASA status. Regarding anatomic severity, Lund et al. [21] was the only study to report the AAST (American Association for the Surgery of Trauma) grade, which classifies perforated peptic ulcer severity based on anatomic findings. The drain group had a mean AAST grade of 3.6, and the no-drain group had a mean of 3.7. No other studies reported the AAST grade.

### 3.3. Meta-Analysis Results

#### 3.3.1. Primary Outcomes

**Figure 2:**
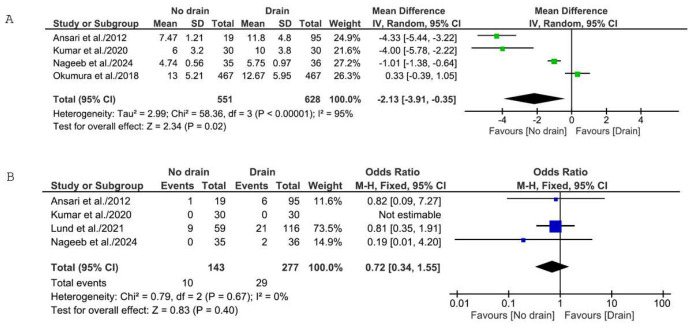
Forest plots for primary outcomes: (**A**) Forest plot comparing LOS between the no-drain group and the control group. (**B**) Forest plot comparing postoperative leak rate between the no-drain group and the control group. SD = standard deviation; IV = inverse variance; CI = confidence interval; M-H = Mantel–Haenszel [21,22,23,24,25].

Pooled analysis on four studies reporting LOS for 1179 patients (551 no-drain, 628 drain) demonstrated a statistically significant reduction in LOS favoring the no-drain strategy [Mean Difference (MD): −2.13 days; 95% Confidence Interval (CI): −3.91 to −0.35; *p* = 0.02], as shown in Figure 2A. However, given the substantial heterogeneity observed across studies (I^2^ = 95%), leave-one-out sensitivity analysis was performed to assess the influence of individual studies on the pooled estimate. After removal of Okumura et al., the pooled estimate remained statistically significant [MD: −3.05 days; 95% CI: −5.62 to −0.47; *p* = 0.02], with heterogeneity remaining high (I^2^ = 95%), while removal of any of the other three studies showed loss of statistical significance with considerable heterogeneity (I^2^ = 96% in all cases). These findings indicate that, when considering LOS, the three RCTs collectively favor drain omission, while the large observational study exerts a heavier weight and moderates the overall estimate. Furthermore, it should be noted that the definition of LOS varied across the included studies. Ansari et al. defined LOS as “time of discharge” from admission, Kumar et al. and Okumura et al. reported “postoperative stay” (calculated from the day of surgery to discharge), while Nageeb et al. [22] reported “hospital stay” without further specification. This variability in outcome definitions represents a source of clinical heterogeneity that may have influenced the pooled estimates and should be considered when interpreting the findings.

Four studies reported data on postoperative leak, comprising 420 patients (143 no-drain, 277 drain) (Figure 2B). Kumar et al. reported zero events in both groups, and the result was not estimable. Pooled analysis using a random-effects model showed no significant difference in leak rates between the no-drain and drain groups [OR: 0.75; 95% CI: 0.35 to 1.61; *p* = 0.45; I^2^ = 0%]. A fixed-effects model yielded similar results [OR: 0.72; 95% CI: 0.34 to 1.55; *p* = 0.40].

#### 3.3.2. Secondary Outcomes

**Figure 3:**
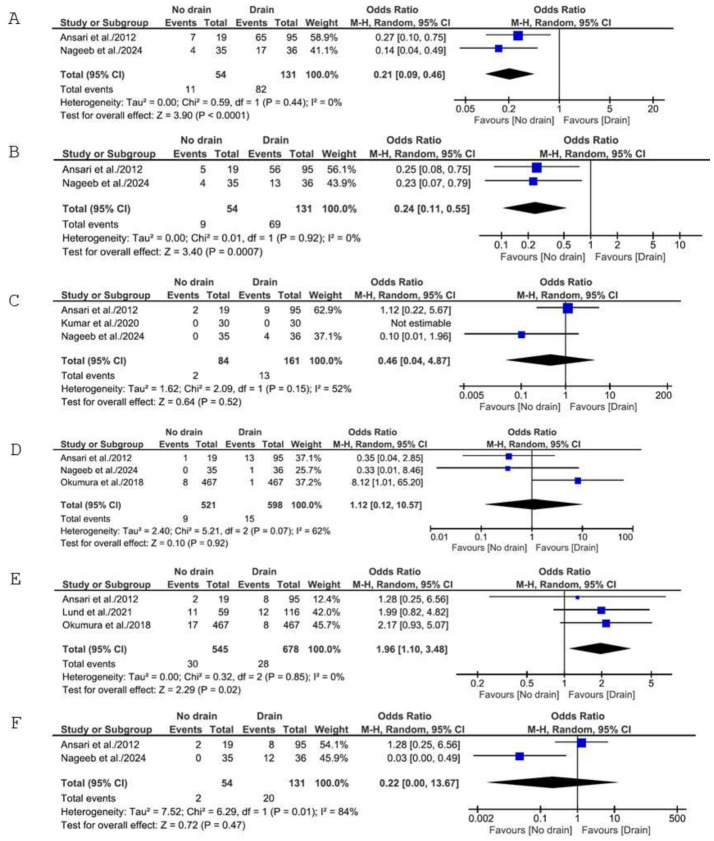
Forest plots for secondary outcomes: (**A**) Forest plot comparing SSIs between the no-drain group and control group. (**B**) Forest plot comparing superficial SSIs between the no-drain group and the control group. (**C**) Forest plot comparing deep SSIs between the no-drain group and the control group. (**D**) Forest plot comparing reoperation rates between the no-drain group and the control group. (**E**) Forest plot comparing postoperative mortality between the no-drain group and the control group. (**F**) Forest plot comparing respiratory complications between the no-drain group and the control group. M-H = Mantel–Haenszel; CI = confidence interval [21,22,23,24,25].

Two studies reported overall SSI rates, including 185 patients (54 no-drain, 131 drain). Pooled analysis demonstrated a statistically significant reduction in SSIs in favor of the no-drain strategy [OR: 0.21; 95% CI: 0.09 to 0.46; *p* < 0.0001; I^2^ = 0%] (Figure 3A). Fixed-effects analysis confirmed this finding [OR: 0.20; 95% CI: 0.09 to 0.45; *p* < 0.0001].

Pooled analysis of superficial SSI rates showed significantly lower odds of superficial SSI for the no-drain group [OR: 0.24; 95% CI: 0.11 to 0.55; *p* = 0.0007; I^2^ = 0%] (Figure 3B). Fixed-effects analysis was identical.

In Ansari et al.’s work, we considered frank intra-abdominal sepsis as deep SSIs. Pooled analysis of the revealed no significant difference between groups [OR: 0.46; 95% CI: 0.04 to 4.87; *p* = 0.52; I^2^ = 52%] (Figure 3C).

Pooled analysis on the three studies reporting reoperation rate for 1119 patients (521 no-drain, 598 drain) showed no significant difference between groups [OR: 1.12; 95% CI: 0.12 to 10.57; *p* = 0.92] (Figure 3D). Due to substantial heterogeneity (I^2^ = 62%), a leave-one-out analysis was performed. After the removal of Okumura et al., heterogeneity resolved completely (I^2^ = 0%) and the pooled estimate shifted to favor no-drain, though not significantly [OR: 0.35; 95% CI: 0.06 to 2.01; *p* = 0.24]. Removing any of the other studies did not favor either group or showed a reduction in heterogeneity.

Three studies reported postoperative mortality, including 1223 patients (545 no-drain, 678 drain). Pooled analysis demonstrated a statistically significant increase in mortality in the no-drain group compared to drain placement [OR: 1.96; 95% CI: 1.10 to 3.48; *p* = 0.02; I^2^ = 0%] (Figure 3E). Fixed-effects analysis yielded identical results.

Pooled analysis on two studies reporting respiratory complications in 185 patients (54 no-drain, 131 drain) showed no significant difference between groups [OR: 0.22; 95% CI: 0.00 to 13.67; *p* = 0.47; I^2^ = 84%] (Figure 3F).

Drain-related complications were reported in two studies. Ansari et al. reported the following events: pain (89.5%), peritubal discharge (36.8%), kinking of the tube (8.4%), drain blockage (22.1%), accidental removal of the tube (6.3%), and restriction of mobility (97.9%). Nageeb et al. [22] reported a 19.4% rate of drain-related infections.

#### 3.3.3. Subgroup Analysis: RCTs

To explore potential bias introduced by observational studies, a subgroup analysis was performed, limited to the three RCTs.

**Figure 4:**
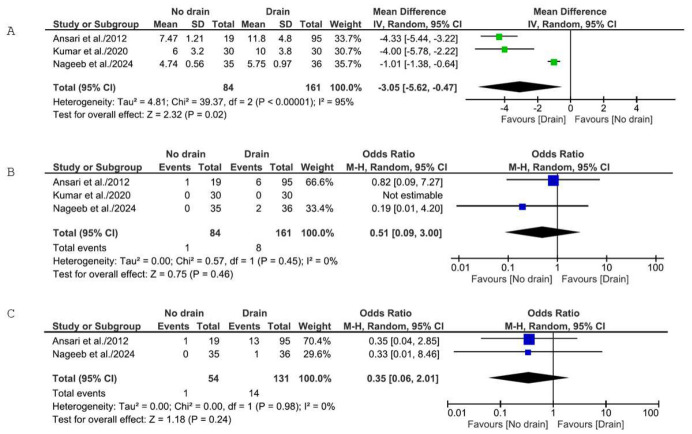
Forest plots comparing outcomes exclusively for RCTs: (**A**) Forest plot comparing LOS between the no-drain group and the control group. (**B**) Forest plot comparing postoperative leak rate between the no-drain group and the control group. (**C**) Forest plot comparing reoperation rates between the no-drain group and the control group. SD = standard deviation; IV = inverse variance; CI = confidence interval; M-H = Mantel–Haenszel [22,24,25].

Pooled analysis on three RCTs including 245 patients (84 no-drain, 161 drain) using a random-effects model demonstrated a statistically significant reduction in LOS favoring no-drain [MD: −3.05 days; 95% CI: −5.62 to −0.47; *p* = 0.02; I^2^ = 95%] (Figure 4A). Removing Nageeb et al. led to increased significance [MD: −4.24 days; 95% CI: −5.18 to −3.30; *p* < 0.00001] and a complete resolution of heterogeneity (I^2^ = 0%). Removal of any of the other RCTs led to a loss of significance, maintaining high heterogeneity. These findings mirror the overall analysis: the cumulative effect of all three RCTs is required to achieve statistical significance.

Of the three RCTs, Kumar et al. [25] had zero leaks and was not estimable for analysis. Pooled analysis of the remaining two RCTs showed no significant difference between groups [OR: 0.51; 95% CI: 0.09 to 3.00; *p* = 0.46; I^2^ = 0%] (Figure 4B). Fixed-effects analysis yielded similar results.

Two RCTs reported reoperation. Pooled analysis showed no significant difference between groups [OR: 0.35; 95% CI: 0.06 to 2.01; *p* = 0.24; I^2^ = 0%] (Figure 4C). Fixed-effects analysis achieved the same results.

### 3.4. Risk of Bias

**Figure 5:**
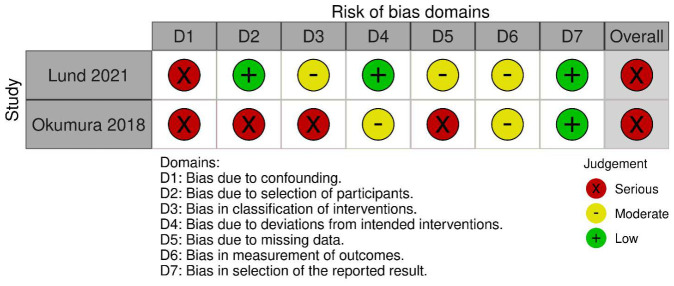
ROBINS-I V2 traffic-light plot [17].

**Figure 6:**
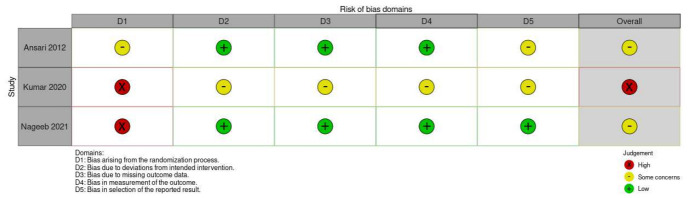
RoB2 traffic-light plot [16].

The risk of bias assessment was performed using the Cochrane RoB 2.0 tool for RCTs and the ROBINS-I V2 tool for non-RCTs. A summary of the risk of bias judgements is presented in Figure 5 and Figure 6.

Among the three included RCTs, two studies demonstrated some concerns regarding the risk of bias. The three studies had limitations primarily in terms of blinding, which is inherent to surgical trials comparing drain omission. The RCT by Kumar et al. [25] was judged to have a high risk of bias, also due to overall bias in incomplete outcome reporting. Ansari et al. [24] had further limitations in the selection of reported outcomes.

The two retrospective cohort studies by Lund et al. [21] and Okumura et al. [23] were assessed using the ROBINS-I tool, and both demonstrated a serious risk of bias. The observational design inherently introduces potential confounding by indication, as the decision to place a drain may be influenced by patient acuity, surgeon preference, or intra-operative findings. Notably, Okumura et al. [23] required PSM to address significant baseline differences between groups, suggesting the presence of unmeasured confounders that could not be fully adjusted for in the analysis.

Across all studies, several limitations were identified that may affect the certainty of the pooled estimates. These include incomplete outcome reporting, particularly regarding surgical technique details, drain management protocols, and patient characteristics. Additionally, the lack of standardized definitions for outcomes such as LOS, prolonged ileus, and intra-abdominal collections precluded meta-analyses for several clinically important endpoints. These methodological limitations should be considered when interpreting the findings of this review.

The GRADE assessment revealed that the certainty of evidence ranged from low to very low across all outcomes, primarily due to the risk of bias in the included studies, inconsistency in effect estimates, indirectness of outcome measures, and imprecision of pooled estimates (Table S1 in Supplementary Materials). Low-certainty evidence suggests that a no-drain strategy may reduce the risk of surgical site infections, with stronger evidence for superficial SSIs. However, evidence for LOS, postoperative leak, reoperation, and mortality was of very low certainty, precluding definitive conclusions. The finding of increased mortality in the no-drain group should be interpreted with particular caution, given the very low certainty and high likelihood of confounding by indication in the observational studies contributing to this outcome.

A subgroup GRADE analysis limited to the RCTs demonstrated very-low-certainty evidence for all outcomes evaluated (Table S2 in Supplementary Materials). LOS was significantly reduced in the no-drain group, but the certainty was very low due to the serious risk of bias, inconsistency, indirectness, and imprecision. Postoperative leak showed no difference between groups, with very low certainty attributable to the serious risk of bias, indirectness, and imprecision. Reoperation rates similarly demonstrated no significant difference, with very low certainty for the same reasons.

### 3.5. Publication Bias

Assessment of publication bias using funnel plots was planned; however, with fewer than 10 studies included in any individual meta-analysis, formal evaluation of funnel plot asymmetry was not performed, as the power of statistical tests is too low to distinguish chance from real asymmetry [28]. Therefore, publication bias could not be reliably assessed and remains an unmeasured potential limitation of this review.

## 4. Discussion

To the best of our knowledge, this is the first systematic review and meta-analysis specifically examining the role of prophylactic intra-abdominal drain placement following primary repair of PPU. Our pooled analysis of five comparative studies, comprising 1354 patients, suggests that a no-drain strategy may be associated with a shorter LOS and a lower rate of superficial surgical site infections, without a demonstrable increase in repair leak, deep SSIs, or reoperation rates. However, an unexpected finding of increased postoperative mortality in the no-drain group emerged from the analysis, which requires careful and nuanced interpretation, given the substantial methodological limitations of the available evidence.

The observed reduction in LOS favoring the no-drain group is consistent with evidence from other abdominal surgical fields. Prophylactic drains have been increasingly questioned in pancreatic and colorectal surgery, where their routine use has been linked to prolonged hospitalization, increased patient discomfort, and drain-related infectious complications without demonstrated benefit in terms of reduced leak or reoperation rates [11,12]. The present findings suggest that a similar paradigm may apply to PPU repair. Drain-related complications reported by Ansari et al. [24] are particularly illustrative in this regard: pain was recorded in 89.5% of drained patients, restriction of mobility in 97.9%, and drain blockage in 22.1%, all of which are plausible contributors to prolonged recovery and extended hospitalization. The substantial drain-related infection rate of 19.4% reported by Nageeb et al. [22] further underscores the potential for the drain itself to serve as a cause for morbidity rather than its prevention.

The reduction in superficial SSIs observed in the no-drain group is similarly consistent with the hypothesis that drain exit sites may provide a portal of entry for cutaneous pathogens, directly contributing to wound infections. This finding was robust across both studies reporting this outcome and was corroborated by the broader SSI analysis. Notably, no significant difference was observed in deep SSI rates, intra-abdominal collections, or leak rates, suggesting that drain omission does not appear to compromise the ability to manage deeper septic complications. Whether this reflects the adequacy of thorough peritoneal lavage as an alternative strategy or the relatively low-risk populations selected across studies remains unclear.

The mortality finding warrants particularly careful interpretation. Pooled analysis of three studies demonstrated a statistically significant increase in 30-day mortality in the no-drain group compared to the drain group. However, this result cannot be disentangled from the profound clinical and methodological heterogeneity of the contributing studies. Two of the three were retrospective in design, rated as carrying a serious risk of bias according to the ROBINS-I tool and, critically, reported no exclusion criteria for perforation size or location. Their populations, therefore, may have included a larger proportion of high-acuity patients with larger perforations, frank peritoneal contamination, and greater hemodynamic compromise, precisely the clinical scenarios in which the presence or absence of a drain may carry the most meaningful prognostic consequences. By contrast, all three RCTs specifically excluded high-risk patients (i.e., ASA III–IV, presentation beyond 48–72 h, frank pyoperitoneum, perforation size greater than 2 cm, or hemodynamic instability), effectively limiting their populations to lower-acuity cases where the impact of drain omission on mortality would be expected to be negligible, and where no mortality signal was indeed observed. The mortality finding of this meta-analysis, therefore, most plausibly reflects the inclusion of a more complex patient substrate in the retrospective studies rather than a true effect of drain omission and should not be interpreted as causal until evaluated in prospective trials designed to encompass the full clinical spectrum of PPU.

The heterogeneity observed across nearly all outcomes extends beyond the mortality finding and represents a central limitation of the entire review. The included studies span four countries, a period of over 25 years, and institutional sizes ranging from a single-center series of 60 patients to a nationwide database of over 900, with fundamental differences in case selection, operative strategy, and postoperative protocols inevitably generating divergent absolute outcome values across virtually all endpoints. The degree of statistical inconsistency is the quantitative expression of this clinical reality. Formal meta-regression to explore heterogeneity sources was considered but not performed: with only four studies contributing to the LOS analysis, meta-regression would require a minimum of approximately ten studies per covariate to produce reliable estimates, and its application to fewer studies is widely recognized to yield unstable and potentially misleading results [28]. Instead, the principal sources of heterogeneity can be identified through structured qualitative analysis. Patient selection differed fundamentally: the three RCTs applied strict exclusion criteria (ASA III–IV, perforation size >2 cm, presentation beyond 48–72 h, frank pyoperitoneum, hemodynamic instability), effectively restricting their populations to low-acuity cases, while the two retrospective studies enrolled broader populations without these restrictions. The surgical approach also varied and was not uniformly reported. Finally, LOS definitions differed substantially: Ansari et al. measured total time from admission to discharge, Kumar et al. [25] and Okumura et al. [23] reported postoperative stay from the day of surgery, and Nageeb et al. [22] reported hospital stay without specification. These definitional inconsistencies mean that the pooled LOS estimate represents an approximation across different metrics rather than a precisely measured effect, contributing directly to the indirectness rating in our GRADE assessment.

These results, while requiring cautious interpretation, should nonetheless serve to stimulate much-needed discussion about the routine use of prophylactic drains in PPU repair. Current surgical practice remains highly variable and largely driven by surgeon habits, institutional tradition, or individual clinical judgment rather than robust evidence [29]. The present analysis, despite its limitations, provides the first quantitative synthesis of the available data and highlights that drain omission is a practice that merits serious scientific scrutiny. It is hoped that this review will influence the design and implementation of adequately powered, prospective, multi-center randomized controlled trials that can definitively address this question with the methodological rigor it deserves.

An important limitation common to all included studies is the incomplete reporting of several preoperative and intra-operative variables that are well-established determinants of outcome in PPU. Perforation size and location are among the most critical prognostic variables in PPU surgery, with larger perforations and gastric localizations generally associated with higher rates of repair failure, postoperative leak, and mortality [30,31,32]. Yet only two of five studies reported perforation size, and only one provided systematic data on the perforation site. The prognostic significance of these variables is not merely academic: a drain may indeed be more justifiable in the context of a large gastric perforation with established peritonitis than in a small duodenal ulcer with minimal contamination. The inability to perform subgroup analyses stratified by these variables represents a major gap in the current evidence base.

Surgeon and institutional experience may represent a relevant modifier of outcomes specifically in the context of laparoscopic PPU repair, where a significant learning curve has been documented, and high-volume centers have been associated with lower conversion rates and reduced postoperative mortality [33,34]. None of the included studies reported data on hospital volume or surgeon caseload, and given that two studies included both open and laparoscopic approaches, the generalizability of the present findings to different practice settings remains uncertain. The learning curve associated with laparoscopic PPU repair is well documented [35], and the decision to omit a drain may carry different risk profiles in the hands of experienced laparoscopic surgeons operating in high-volume centers compared to those of surgeons with limited exposure to this procedure in lower-volume settings. Institutional infrastructure for postoperative monitoring and early detection of complications (including access to computed tomography, interventional radiology, and intensive care) may further mediate the consequences of drain omission, as the ability to detect and manage an intra-abdominal collection percutaneously may reduce the clinical impact of not having a drain in situ. Hospital volume has been shown to influence outcomes in PPU repair [34], and its omission from study reporting limits the generalizability and clinical applicability of the present findings.

The overall certainty of evidence, rated very low to low for all outcomes using the GRADE framework, reflects the cumulative weight of these limitations: risk of bias, inconsistency, indirectness, and imprecision. In practical terms, this means that future adequately designed studies are very likely to have important impacts on estimates of effect and may change conclusions substantially. Any clinical extrapolation from the present findings should be approached with commensurate humility.

This review has several strengths worth acknowledging. It follows a prospectively registered protocol, adheres to PRISMA guidelines, applies a validated risk of bias assessment framework, and employs appropriate methods for handling statistical heterogeneity, including sensitivity and subgroup analyses. The inclusion of a propensity-score matched cohort from Okumura et al. rather than the full unmatched cohort represents an attempt to minimize selection bias in the analysis.

Several limitations also deserve explicit acknowledgment. The small number of eligible studies precluded formal assessment of publication bias, formal meta-regression analysis, and limited the power of most subgroup analyses. The anticipated subgroup analyses by surgical approach (laparoscopic versus open), perforation site, and patient risk profile could not be performed due to missing data. Significant heterogeneity in the definition of outcomes (particularly for prolonged ileus, intra-abdominal collections, and mortality timing) prevented meta-analyses of several clinically important endpoints. The two retrospective studies contributed most of the total patient population, which means that the overall pooled estimates are heavily weighted by designs that are inherently vulnerable to selection bias and unmeasured confounding. Furthermore, data from Okumura et al. derive from a propensity-score matched cohort rather than two independent patient groups. Although PSM reduces observable confounding, residual confounding from variables not captured in the administrative database (including perforation size, degree of peritoneal soiling, and surgeon experience) cannot be excluded, as such, results from this study should be interpreted with corresponding caution.

## 5. Conclusions

This systematic review and meta-analysis suggest that, based on low-certainty evidence, drain omission following the primary repair of PPU may be associated with a shorter hospital stay and fewer superficial SSIs without increasing the risk of leak, deep SSI, or reoperation. However, based on very low-certainty evidence and with a high probability of confounding by indication, a pooled association with increased postoperative mortality was observed in the no-drain group, driven predominantly by two retrospective studies with important methodological limitations. This finding should not be interpreted as causal and precludes any definitive recommendation in favor of drain omission at this time. The results of this analysis should not be viewed as practice-changing but rather as a call to action: the routine use of prophylactic drains in PPU repair has never been subjected to adequately powered, prospective evaluation, and the heterogeneity of current practice reflects this evidentiary void. Future prospective multi-center RCTs, with standardized patient selection criteria, systematic reporting of perforation characteristics, operative details, hospital volume, and surgeon experience, are urgently needed to resolve this question and guide evidence-based decision-making.

## Figures and Tables

**Table 1:** Study and population characteristics. n = number; RCT = randomized controlled trial.

Authors/Year	Study Type		Study Centers, n	Study Country	Study Period	Sample Size	Drain Placement, n	
							Yes	No
Ansari et al./2012 [24]	RCT	Single center	1	India	2009–2010	114	95	19
Kumar et al./2020 [25]	RCT	Single center	1	India	2017–2019	60	30	30
Lund et al./2021 [21]	Retrospective	Single center	1	USA	2008–2019	175	116	59
Nageeb et al./2024 [22]	RCT	Single center	1	Egypt	2022–2024	71	36	35
Okumura et al./2018 [23]	Retrospective	Multi-center	324	Japan	2010–2016	934	467	467

**Table 2:** Characteristics of surgical interventions and exclusion criteria. N/A = not available, PPU = perforated peptic ulcer.

Authors/Year	Surgery	Approach	Type of Drain	Exclusion Criteria
Ansari et al./2012 [24]	Graham patch	Open	Sub-hepatic and/or pelvic	Critically sick patients (pulse rate >100/min, blood pressure <90 mm Hg (systolic), deranged arterial blood gases or deranged renal/cardio-pulmonary function), patients presenting after 72 h of onset of symptoms, past history of similar illness, perforation >2 cm in size, frank pyoperitoneum (presence of frank yellow pus in the peritoneal cavity), patients who died within 24 h of emergency laparotomy
Kumar et al./2020 [25]	Graham patch	Open	28 Fr	Multiple perforations, traumatic perforations and severe co-morbid conditions
Lund et al./2021 [21]	Graham patch, modified Graham patch, gastric resection, serosal patch	Open or laparoscopic	N/A	Traumatic perforations, perforations secondary to anastomotic ulcer, patients with index operations outside of index center
Nageeb et al./2024 [22]	Graham patch	Open	Subhepatic and pelvic	ASA score III and IV, the symptoms onset was longer than 48 h, purulent peritonitis (presence of frank pus), ulcer size larger than 20 mm or suspicious malignancy, sealed off perforation, more than one perforation, presence of bleeding ulcer and who underwent additional procedures
Okumura et al./2018 [23]	Gastric/duodenal suturing, including Graham patch	Open or laparoscopic	N/A	Confirmed diagnosis of neoplasm, iatrogenic perforation, traumatic perforation, perforation caused by foreign bodies, pathogeneses other than peptic perforations, and patients who underwent PPU repair after 8 or more days from the admission date.

**Table 3:** Patient characteristics. SD = standard deviation; N/A = not available.

	Drain Status	Population	Age (Years), Mean ± SD	Gender
				Male
Ansari et al./2012 [24]	Yes	95	45.11 ± 16.93	15
	No	19	44.94 ± 37.5	4
Kumar et al./2020 [25]	Yes	30	N/A	29
	No	30	N/A	29
Lund et al./2021 [21]	Yes	116	N/A	N / A
	No	59	N/A	N / A
Nageeb et al./2024 [22]	Yes	36	41.56 ± 14.34	31
	No	35	36.77 ± 7.54	34
Okumura et al./2018 [23]	Yes	467	N/A	353
	No	467	N/A	353

## Data Availability

The data presented in this study are available on request from the corresponding author.

## Supplementary Materials

The following supporting information can be downloaded at: https://www.mdpi.com/article/10.3390/medicina62051003/s1, Table S1: GRADE assessment for all results; Table S2: GRADE assessment for results of RCTs. Reference [36] is cited in the supplementary materials.

## Abbreviations

The following abbreviations are used in this manuscript:

PPU	Perforated peptic ulcer
PRISMA	Preferred Reporting Items for Systematic Reviews and Meta-Analyses
LOS	Length of hospital stay
SSI	Surgical site infection
OR	Odds ratio
CI	Confidence interval
MD	Mean difference
RCT	Randomized controlled trial
PUD	Peptic ulcer disease
PPI	Proton pump inhibitor
PROSPERO	International Prospective Register of Systematic Reviews
PICOS	Population, Intervention, Comparator, Outcome, Study design
CDC	Centers for Disease Control and Prevention
RoB 2.0	Cochrane Risk of Bias tool, version 2
ROBINS-I	Risk Of Bias In Non-randomized Studies of Interventions
GRADE	Grading of Recommendations, Assessment, Development and Evaluations
GDT	GRADE Development Tool
SoF	Summary of Findings
IQR	Interquartile range
SD	Standard deviation
ASA	American Society of Anesthesiologists
AAST	American Association for the Surgery of Trauma
PSM	Propensity-score matching
IV	Inverse variance
M-H	Mantel–Haenszel
