# Supplementary material for: Perioperative Outcomes of No-Drain Strategy in Primary Repair of Perforated Peptic Ulcer: A Systematic Review and Meta-Analysis
Source: Medicina (Kaunas). 2026 May 21;62(5):1003. doi: 10.3390/medicina62051003 (PMC13209119; doi:10.3390/medicina62051003)
Supplement: Supplementary file 1 [file medicina-62-01003-s001.zip › PRISMA 2020 Checklist.pdf]

# PRISMA 2020 Checklist

| Section and Topic | Item # | Checklist item | Location where item is reported |
|---|---|---|---|
| **TITLE** | | | |
| Title | 1 | Identify the report as a systematic review. | Title: "Perioperative outcomes of no-drain strategy in primary repair of perforated peptic ulcer: a systematic review and meta-analysis" |
| **ABSTRACT** | | | |
| Abstract | 2 | See the PRISMA 2020 for Abstracts checklist. | Abstract (Background, Methods, Results, Conclusions sections) |
| **INTRODUCTION** | | | |
| Rationale | 3 | Describe the rationale for the review in the context of existing knowledge. | Introduction, paragraphs 3–6 (discussion of PPU burden, current drain practice, and evidence gap |
| Objectives | 4 | Provide an explicit statement of the objective(s) or question(s) the review addresses. | Introduction, final paragraph: "The objective of this study was to compare the perioperative outcomes of a no-drain strategy versus routine drain placement..." |
| **METHODS** | | | |
| Eligibility criteria | 5 | Specify the inclusion and exclusion criteria for the review and how studies were grouped for the syntheses. | Inclusion criteria (PICOS framework); exclusion criteria |
| Information sources | 6 | Specify all databases, registers, websites, organisations, reference lists and other sources searched or consulted to identify studies. Specify the date when each source was last searched or consulted. | Search strategy: MEDLINE/PubMed, Web of Science, Cochrane Library, Scopus; last search 22 February 2026; PROSPERO registration noted |
| Search strategy | 7 | Present the full search strategies for all databases, registers and websites, including any filters and limits used. | Search strategy: full search strings for all four databases presented; date limits (January 2010–February 2026) stated |
| Selection process | 8 | Specify the methods used to decide whether a study met the inclusion criteria of the review, including how many reviewers screened each record and each report retrieved, whether they worked independently, and if applicable, details of automation tools used in the process. | Two independent reviewers (LDA, AM) screened titles/abstracts; third reviewer (MZ) resolved conflicts; Rayyan software used |
| Data collection process | 9 | Specify the methods used to collect data from reports, including how many reviewers collected data from each report, whether they worked independently, any processes for obtaining or confirming data from study investigators, and if applicable, details of automation tools used in the process. | Data extraction: two independent reviewers (LDA, AM) using a predesigned extraction form; disagreements resolved by third reviewer (MZ) |
| Data items | 10a | List and define all outcomes for which data were sought. Specify whether all results that were compatible with each outcome domain in each study were sought (e.g. for all measures, time points, analyses), and if not, the methods | Outcomes: primary outcomes (LOS, leak) and secondary outcomes |

# PRISMA 2020 Checklist

| Section and Topic | Item # | Checklist item | Location where item is reported |
|---|---|---|---|
| | | used to decide which results to collect. | (morbidity, mortality, SSI, ileus, reoperation, drain complications) defined |
| | 10b | List and define all other variables for which data were sought (e.g. participant and intervention characteristics, funding sources). Describe any assumptions made about any missing or unclear information. | Data extraction: study characteristics, patient characteristics (age, sex, Boey/AAST score, ASA, perforation site/size, time to surgery, shock), intervention details listed |
| Study risk of bias assessment | 11 | Specify the methods used to assess risk of bias in the included studies, including details of the tool(s) used, how many reviewers assessed each study and whether they worked independently, and if applicable, details of automation tools used in the process. | RoB 2.0 for RCTs; ROBINS-I V2 for observational studies |
| Effect measures | 12 | Specify for each outcome the effect measure(s) (e.g. risk ratio, mean difference) used in the synthesis or presentation of results. | Data analysis: OR for dichotomous outcomes, MD for continuous outcomes |
| Synthesis methods | 13a | Describe the processes used to decide which studies were eligible for each synthesis (e.g. tabulating the study intervention characteristics and comparing against the planned groups for each synthesis (item #5)). | Meta-analysis performed if ≥2 studies reported data for a given outcome |
| | 13b | Describe any methods required to prepare the data for presentation or synthesis, such as handling of missing summary statistics, or data conversions. | Median/IQR converted to mean/SD using Wan formulae; multiple group data combined using Cochrane formulae |
| | 13c | Describe any methods used to tabulate or visually display results of individual studies and syntheses. | Tables 1–3 (study/patient characteristics); forest plots (Figures 2–4) |
| | 13d | Describe any methods used to synthesize results and provide a rationale for the choice(s). If meta-analysis was performed, describe the model(s), method(s) to identify the presence and extent of statistical heterogeneity, and software package(s) used. | Random-effects model (DerSimonian-Laird); Cochran Q and $I^2$ for heterogeneity; RevMan 5.4 used |
| | 13e | Describe any methods used to explore possible causes of heterogeneity among study results (e.g. subgroup analysis, meta-regression). | Subgroup analysis planned (study design, surgical approach, perforation site, patient risk profile); meta-regression not performed (insufficient studies) |
| | 13f | Describe any sensitivity analyses conducted to assess robustness of the synthesized results. | Fixed-effects model for low-moderate heterogeneity; leave-one-out analysis for high heterogeneity ($I^2$ >50%) |
| Reporting bias assessment | 14 | Describe any methods used to assess risk of bias due to missing results in a synthesis (arising from reporting biases). | Funnel plots planned but not feasible (<10 studies per outcome); explicitly acknowledged as limitation |
| Certainty | 15 | Describe any methods used to assess certainty (or confidence) in the body of evidence for an outcome. | GRADE framework; GRADEpro GDT |

# PRISMA 2020 Checklist

| Section and Topic | Item # | Checklist item | Location where item is reported |
|---|---|---|---|
| assessment | | | software; Summary of Findings table constructed |
| **RESULTS** | | | |
| Study selection | 16a | Describe the results of the search and selection process, from the number of records identified in the search to the number of studies included in the review, ideally using a flow diagram. | 301 records identified; 5 studies included; PRISMA flow diagram (Figure 1) |
| | 16b | Cite studies that might appear to meet the inclusion criteria, but which were excluded, and explain why they were excluded. | 3 studies excluded: absence of a comparator group and absence of outcomes of interest. |
| Study characteristics | 17 | Cite each included study and present its characteristics. | Tables 1–3; narrative summary of study design, country, period, sample size, surgical technique, patient characteristics |
| Risk of bias in studies | 18 | Present assessments of risk of bias for each included study. | RoB 2.0 and ROBINS-I V2 results summarised; Figures S1–S2 (traffic-light plots in Supplementary) |
| Results of individual studies | 19 | For all outcomes, present, for each study: (a) summary statistics for each group (where appropriate) and (b) an effect estimate and its precision (e.g. confidence/credible interval), ideally using structured tables or plots. | Individual study event counts/means reported within text and forest plots |
| Results of syntheses | 20a | For each synthesis, briefly summarise the characteristics and risk of bias among contributing studies. | Results 3.3 (per-outcome narrative of contributing studies); Results 3.4 (risk of bias summary) |
| | 20b | Present results of all statistical syntheses conducted. If meta-analysis was done, present for each the summary estimate and its precision (e.g. confidence/credible interval) and measures of statistical heterogeneity. If comparing groups, describe the direction of the effect. | Results 3.3.1–3.3.3 |
| | 20c | Present results of all investigations of possible causes of heterogeneity among study results. | Results 3.3: leave-one-out analyses; qualitative sources identified (patient selection, LOS definitions, study design) |
| | 20d | Present results of all sensitivity analyses conducted to assess the robustness of the synthesized results. | Fixed-effects sensitivity analyses and leave-one-out analyses reported for each outcome |
| Reporting biases | 21 | Present assessments of risk of bias due to missing results (arising from reporting biases) for each synthesis assessed. | Funnel plot assessment not possible (<10 studies); acknowledged as unmeasured limitation |
| Certainty of evidence | 22 | Present assessments of certainty (or confidence) in the body of evidence for each outcome assessed. | GRADE ratings (very low to low) per outcome; narrative summary in text |
| **DISCUSSION** | | | |
| Discussion | 23a | Provide a general interpretation of the results in the context of other evidence. | Results interpreted against drain |

# PRISMA 2020 Checklist

| Section and Topic | Item # | Checklist item | Location where item is reported |
|---|---|---|---|
| | | | evidence from pancreatic/colorectal surgery; drain-related complication data contextualised |
| | 23b | Discuss any limitations of the evidence included in the review. | Heterogeneity, incomplete outcome reporting, missing perforation characteristics, surgeon/volume effects, GRADE limitations |
| | 23c | Discuss any limitations of the review processes used. | Small number of studies; publication bias not assessable; subgroup analyses not feasible; meta-regression not performed |
| | 23d | Discuss implications of the results for practice, policy, and future research. | Call for adequately powered multicenter RCTs; current results insufficient to change practice |
| **OTHER INFORMATION** | | | |
| Registration and protocol | 24a | Provide registration information for the review, including register name and registration number, or state that the review was not registered. | PROSPERO registration CRD420251053099, registered 14 May 2025. |
| | 24b | Indicate where the review protocol can be accessed, or state that a protocol was not prepared. | Protocol accessible through PROSPERO |
| | 24c | Describe and explain any amendments to information provided at registration or in the protocol. | No amendments. |
| Support | 25 | Describe sources of financial or non-financial support for the review, and the role of the funders or sponsors in the review. | Funding section: "This research received no external funding"; Acknowledgements: partially supported by Italian Ministry of Health – Ricerca Corrente 2027 |
| Competing interests | 26 | Declare any competing interests of review authors. | No conflicts of interest. |
| Availability of data, code and other materials | 27 | Report which of the following are publicly available and where they can be found: template data collection forms; data extracted from included studies; data used for all analyses; analytic code; any other materials used in the review. | Data available on request from corresponding author |

*From:* Page MJ, McKenzie JE, Bossuyt PM, Boutron I, Hoffmann TC, Mulrow CD, et al. The PRISMA 2020 statement: an updated guideline for reporting systematic reviews. BMJ 2021;372:n71. doi: 10.1136/bmj.n71. This work is licensed under CC BY 4.0. To view a copy of this license, visit https://creativecommons.org/licenses/by/4.0/
