# Supplementary material for: Perioperative Outcomes of No-Drain Strategy in Primary Repair of Perforated Peptic Ulcer: A Systematic Review and Meta-Analysis
Source: Medicina (Kaunas). 2026 May 21;62(5):1003. doi: 10.3390/medicina62051003 (PMC13209119; doi:10.3390/medicina62051003)
Supplement: Supplementary file 1 [file medicina-62-01003-s001.zip › Tables S1 and S2.pdf]

Table S1. GRADE assessment for all results. PPU = perforated peptic ulcer; CI = confidence interval; LOS = length of stay; RCT = randomized controlled trial; MD = mean difference; OR = odds ratio.

No drain compared to Drain for Primary repair after PPU

Certainty assessment							Summary of findings				
Participants (studies) Follow-up	Risk of bias	Inconsistency	Indirectness	Imprecision	Publication bias	Overall certainty of evidence	Study event rates (%)		Relative effect (95% CI)	Anticipated absolute effects	
							With Drain	With No drain		Risk with Drain	Risk difference with No drain
**Lenght Of Stay (LOS)**											
1179 (4 RCTs)	very serious[a]	very serious[b]	serious[c]	serious[d]	none	⊕◯◯◯ Very low[a,b,c,d]	628	551	-	628	MD **2.13 lower** (3.91 lower to 0.35 lower)
**Leak**											
420 (4 RCTs)	very serious[a]	not serious	serious[c]	very serious[e]	none	⊕◯◯◯ Very low[a,c,e]	29/277 (10.5%)	10/143 (7.0%)	**OR 0.72** (0.34 to 1.55)	29/277 (10.5%)	**27 fewer per 1000** (from 66 fewer to 49 more)
**SSIs**											
185 (2 RCTs)	serious[a]	not serious	serious[c,f]	not serious	none	⊕⊕◯◯ Low[a,c,f]	82/131 (62.6%)	11/54 (20.4%)	**OR 0.21** (0.09 to 0.46)	82/131 (62.6%)	**366 fewer per 1000** (from 495 fewer to 191 fewer)
**Superficial SSIs**											
185 (2 RCTs)	serious[a]	not serious	serious[b,f]	not serious	none	⊕⊕◯◯ Low[a,b,f]	69/131 (52.7%)	9/54 (16.7%)	**OR 0.24** (0.11 to 0.55)	69/131 (52.7%)	**316 fewer per 1000** (from 418 fewer to 147 fewer)

No drain compared to Drain for Primary repair after PPU

Certainty assessment							Summary of findings				
**Deep SSIs**											
245 (2 RCTs)	serious[a]	not serious	serious[b,f]	not serious	none	⊕⊕◯◯ Low[a,b,f]	13/161 (8.1%)	2/84 (2.4%)	**OR 0.46** (0.04 to 4.87)	13/161 (8.1%)	**42 fewer per 1000** (from 77 fewer to 219 more)
**Reoperation**											
1119 (3 RCTs)	serious[a]	serious[b]	serious[c]	very serious[e]	none	⊕◯◯◯ Very low[a,b,c,e]	15/598 (2.5%)	9/521 (1.7%)	**OR 1.12** (0.12 to 10.57)	15/598 (2.5%)	**3 more per 1000** (from 22 fewer to 189 more)
**Post op mortality**											
1223 (3 RCTs)	very serious[a]	serious[g]	serious[c]	serious[e]	none	⊕◯◯◯ Very low[a,c,e,g]	28/678 (4.1%)	30/545 (5.5%)	**OR 1.96** (1.10 to 3.48)	28/678 (4.1%)	**37 more per 1000** (from 4 more to 89 more)
**Respiratory complications**											
185 (2 RCTs)	serious[a]	serious[b]	serious[c]	very serious[e]	none	⊕◯◯◯ Very low[a,b,c,e]	20/131 (15.3%)	2/54 (3.7%)	**OR 0.22** (0.00 to 13.67)	20/131 (15.3%)	**115 fewer per 1000** (from -- to 559 more)

CI: confidence interval; **MD:** mean difference; **OR:** odds ratio

Explanations

a. Most information is from studies with at least some concern for bias
b. Minimal overlap, high heterogeneity
c. Different post-operative management protocols
d. Trivial effect
e. High OR CI ratio
f. Different outcome reporting
g. Wide CI

Table S2. GRADE assessment for results of RCTs. PPU = perforated peptic ulcer; RCT = randomized controlled trial; CI = confidence interval; LOS = length of stay; MD = mean difference; OR = odds ratio.

No drain compared to Drain for Primary repair for PPU (RCTs)

Certainty assessment							Summary of findings				
Participants (studies) Follow-up	Risk of bias	Inconsistency	Indirectness	Imprecision	Publication bias	Overall certainty of evidence	Study event rates (%)		Relative effect (95% CI)	Anticipated absolute effects	
							With Drain	With No drain		Risk with Drain	Risk difference with No drain
**LOS**											
245 (3 RCTs)	serious[a]	serious	serious[b]	serious[c]	none	⊕◯◯◯ Very low[a,b,c]	161	84	-	161	MD **3.05 lower** (5.62 lower to 0.47 lower)
**Leak**											
245 (3 RCTs)	serious[a]	not serious	serious[b]	serious[c]	none	⊕◯◯◯ Very low[a,b,c]	8/161 (5.0%)	1/84 (1.2%)	**OR 0.51** (0.09 to 3.00)	8/161 (5.0%)	**24 fewer per 1000** (from 45 fewer to 86 more)
**Reoperation**											
185 (2 RCTs)	serious[a]	not serious	serious[b]	serious[c]	none	⊕◯◯◯ Very low[a,b,c]	14/131 (10.7%)	1/54 (1.9%)	**OR 0.35** (0.06 to 2.01)	14/131 (10.7%)	**67 fewer per 1000** (from 100 fewer to 87 more)

CI: confidence interval; **MD:** mean difference; **OR:** odds ratio

Explanations

a. Most information is from studies with at least some concern for bias
b. Different post-operative management protocols
c. High OR CI ratio
